# Immunoglobulin G4-related disease with multi-organ involvement: A case report

**DOI:** 10.1097/MD.0000000000047096

**Published:** 2026-01-16

**Authors:** Jing Qiao, Xiaoxia Xu, Ze Wang, Ping Chen, Fene Hao, Yinglong Huang

**Affiliations:** a Department of Gastroenterology, Affiliated Hospital of Inner Mongolia Medical University, Hohhot, Inner Mongolia Autonomous Region, China; b Department of Imaging, Affiliated Hospital of Inner Mongolia Medical University, Hohhot, Inner Mongolia Autonomous Region, China.

**Keywords:** autoimmune hepatitis, autoimmune pancreatitis, case report, glucocorticoid, immunoglobulin G4-related disease

## Abstract

**Rationale:**

Immunoglobulin G4-related disease (IgG4-RD) is a rare, chronic, and progressive clinical condition characterized by elevated serum IgG4 levels and significant infiltration of IgG4-positive plasma cells, can affect various organs and tissues. However, multi-organ involvement, particularly in the gastrointestinal tract, has rarely been reported. This case report presents a rare instance of multi-organ IgG4-RD, aiming to raise clinicians’ awareness of the diverse manifestations of this condition, thereby mitigating its potential for misdiagnosis and subsequent unnecessary surgical interventions.

**Patient concerns:**

This case report describes a 76-year-old female presented with a chief complaint of intermittent abdominal pain over the past decade and a 20-day history of jaundice. The laboratory findings revealed elevated serum IgG4 levels 21.5 g/L. Liver function test results show significant abnormalities. Magnetic resonance imaging and contrast-enhanced computed tomography of the upper abdomen indicated biliary duct dilation and narrowing of the pancreatic segment of the common bile duct. Gastroscopy revealed inflammatory changes in the cardia. Gastric fundus elevation indicated gastric antrum elevation in external gallbladder pressure. Histopathological analysis of biopsies from the stomach, pancreas, and liver revealed infiltration by plasma cells, eosinophils, and lymphocytes, with a plasma cell IgG4(+) to IgG(+) cell ratio exceeding 40%.

**Diagnoses:**

Based on the clinical, laboratory, and pathological findings, the patient was diagnosed with IgG4-RD, including IgG4-related pancreatitis (autoimmune pancreatitis), immunoglobulin G4-related sclerosing cholangitis, immunoglobulin G4-related autoimmune hepatitis, and immunoglobulin G4-related gastrointestinal disease.

**Interventions:**

Therapy was initiated on March 8, 2022, with oral prednisone acetate (30 mg once daily) in conjunction with acid suppression and calcium supplementation.

**Outcomes:**

Following glucocorticoid administration, the skin and scleral jaundice showed significant improvement compared to previous assessments. Liver function tests and serum IgG4 levels also demonstrated notable improvement, and subsequent magnetic resonance imaging and gastroscopy examinations revealed further amelioration.

**Lessons:**

IgG4-RD is a rare condition with unclear pathogenesis. Given its diverse clinical presentations and propensity for multi-organ involvement, IgG4-RD is frequently subject to missed or incorrect diagnoses, potentially leading to unnecessary surgical interventions or other therapeutic approaches. The diagnosis and management of this case involving multiple organs aim to enhance clinicians’ understanding of IgG4-RD, thereby facilitating timely administration of the most effective treatment.

## 1. Introduction

Immunoglobulin G4-related disease (IgG4-RD) is a chronic immune-mediated fibroinflammatory condition.^[1]^ Its pathogenesis is multifactorial, involving both genetic and environmental factors, as well as innate and adaptive immunity, autoantigens, and antibodies, along with T- and B-cell interactions.^[2,3]^ The most important and frequently reported pathogenic mechanism of IgG4-RD is an antigen-driven immune response, predominantly mediated by a specific subset of CD4+ T cells.^[2]^ Pathologically, it is characterized by lymphoplasmacytic infiltrates rich in IgG4-positive plasma cells, storiform fibrosis, obliterative phlebitis, and eosinophilic infiltration. The disease can affect nearly all organs, including the pancreas, lacrimal glands, salivary glands, retroperitoneum, biliary tract, kidneys, lungs, and thyroid and pituitary glands, with multi-organ involvement commonly observed.^[4]^ IgG4-RD frequently affects the pancreas and the hepatobiliary system; however, gastrointestinal involvement is rare. Given its diverse clinical presentation, IgG4-RD is often misdiagnosed or overlooked. The diagnosis of IgG4-RD is established through serological, imaging, and histopathological findings.^[5]^ Patients with IgG4-RD disease have a significantly increased risk of malignancy. Corticosteroids are the first-line therapeutic intervention for this condition.^[6,7]^

Here, we present a rare case of IgG4-RD affecting the pancreas, biliary tract, liver, stomach, and ureter, with subsequent disease control achieved through glucocorticoid administration. We reviewed the relevant literature to delineate the diagnostic criteria and characteristic features of IgG4-RD in various organ systems. In this case report, we aim to raise clinicians’ awareness of the diverse clinical presentations of IgG4-RD, thereby reducing the rate of missed or incorrect diagnoses and facilitating the implementation of appropriate therapeutic strategies.

## 2. Case presentation

**Figure 1.**
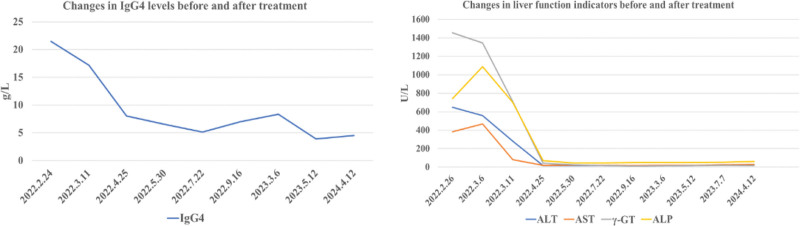
Changes in laboratory parameters before and after treatment. Posttreatment IgG4 and liver function indicators show significant improvement compared to pretreatment levels. γ-GT = gamma-glutamyl transferase, ALT = alanine transaminase, AST = aspartate aminotransferase, ALP = alkaline phosphatase IgG4 = immunoglobulin G4.

**Figure 2.**
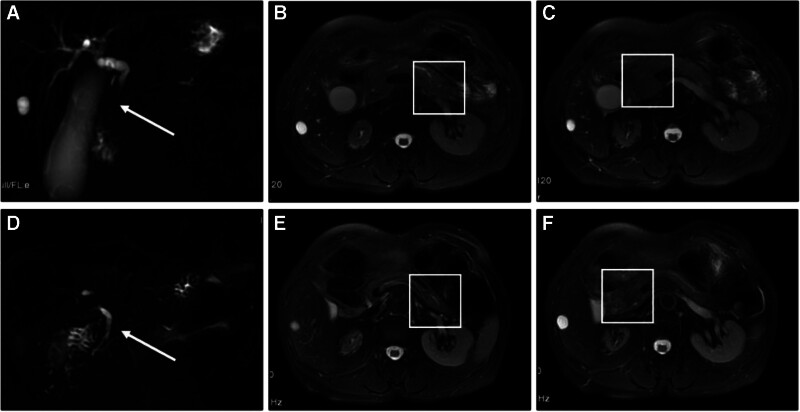
Imaging findings before and after treatment. (A–C) Pretreatment imaging findings. (A) Abdominal MRI showing stenosis of the intrapancreatic common bile duct, and gallbladder enlargement with wall thickening. (B) Abdominal MRI showing enlargement and fullness of the pancreatic body and tail. (C) Abdominal MRI showing enlargement and fullness of the pancreatic head. (D, E) Posttreatment imaging findings. (D) Abdominal MRI showing improvement in the stenosis of the intrapancreatic common bile duct, and a normal-sized gallbladder. (E) Abdominal MRI showing reduction in the fullness of the pancreatic body and tail. (F) Abdominal MRI showing reduction in the fullness of the pancreatic head. MRI = magnetic resonance imaging.

A 76-year-old female presented with a chief complaint of intermittent abdominal pain spanning a decade and a 20-day history of jaundice, prompting admission to our gastroenterology department on February 26, 2022. The patient had experienced epigastric colicky pain for the past 10 years, initially diagnosed as acute pancreatitis at a local hospital. Twenty days prior, the abdominal pain had worsened and was accompanied by fatigue, jaundice, and darkening of the urine. Four days before presentation, the patient visited our hospital’s hepatobiliary surgery outpatient clinic for relevant tests, with the following liver function results: IgG4 level, 21.5 g/L (Fig. 1); and carbohydrate antigen 19-9 (CA 19-9) level, 106.1 U/mL. Liver function test results were as follows: alanine transaminase (ALT) 537.1 U/L, aspartate aminotransferase (AST) 413.4 U/L, gamma-glutamyl transferase (γ-GT) 1086.2 U/L, alkaline phosphatase (ALP) 596.0 U/L, total bilirubin (TBil) 156.5 μmol/L, and direct bilirubin (DBil) 127.9 μmol/L. IgG2 level was 469.0 mg/dL. Erythrocyte sedimentation rate was 36.0 mm/h. Abdominal Doppler ultrasonography revealed a hepatic cyst, mild intrahepatic bile duct dilation, gallbladder enlargement, thickened bile with sediment formation, and dilation of the common bile duct with patency of the visualized segment. The pancreas, particularly the head, was enlarged, with mild dilation of the central pancreatic duct, suggestive of chronic inflammatory changes. Abdominal magnetic resonance imaging (MRI) revealed a small cyst in the right lobe of the liver concurrent with stenosis of the intrapancreatic segment of the common bile duct, cholecystitis, and right renal atrophy (Fig. 2).

**Figure 3.**
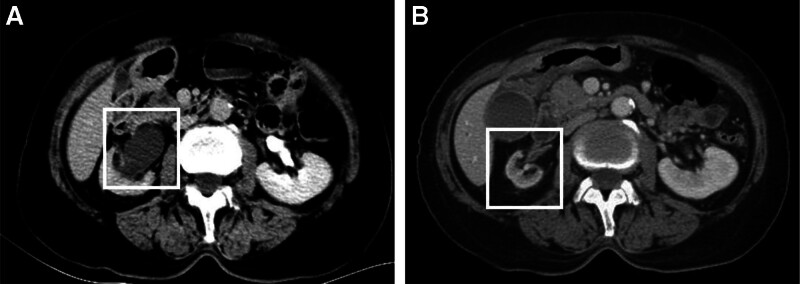
Imaging findings before and after ureteral stent placement. (A) Preplacement: CT urography (CTU) showing wall thickening and luminal narrowing of the right ureter at the level of the iliac vessels, with right hydronephrosis and hydroureter, as well as right renal atrophy with impaired function. (B) Postplacement: abdominal CT showing resolution of right hydronephrosis and hydroureter, along with right renal atrophy. CT = computed tomography, CTU = computed tomography urography.

In 2005, following symptoms of fatigue, weight loss, and dyspepsia, the patient was evaluated at a hospital in Beijing and diagnosed with cecal cancer. The patient underwent surgical resection, followed by 6 cycles of oxaliplatin-based chemotherapy. In 2021, the patient underwent ureteral stent placement and removal due to an inflammatory ureteral stricture (Fig. 3). The patient presented with a history of hypertension and peak blood pressure of 180/140 mm Hg. Management included the administration of oral amlodipine besylate and metoprolol tartrate to control the blood pressure. The patient did not have diabetes or coronary heart disease. The patient’s personal and family histories were unremarkable.

**Figure 4.**
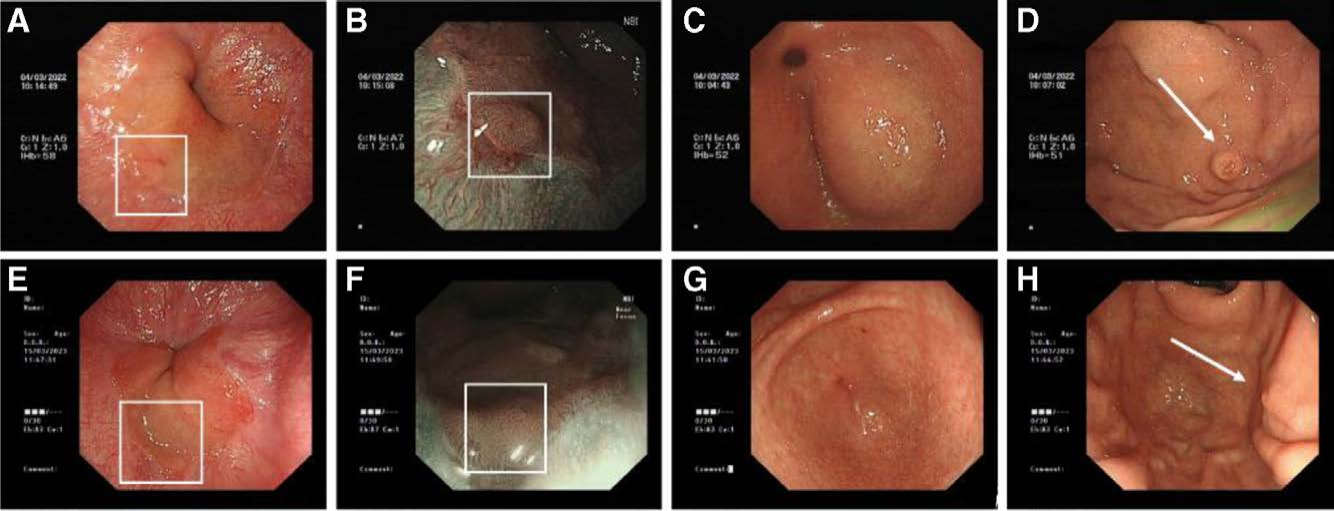
Gastroscopy before and after treatment. (A–D) Gastroscopy before treatment. (A, B) Cardia: rough mucosal bulge near the dentate line. (C) Gastric antrum: a significant external pressure bulge is visible, with a smooth, mucosa-lined surface. (D) Gastric fundus: a hemispherical bulge of approximately 0.6 cm is seen on the side of the anterior wall, with yellowish and erosive mucosa. (E–G) Gastroscopy after treatment. (E, F) Cardia: congestion and edema of the mucosa near the dentate line improved compared to the previous findings. (G) Gastric antrum: significant external pressure bulge is no longer visible. (H) Gastric fundus: an indentation mark is visible on the side of the anterior wall (where the hemispherical yellowish-white bulge is located).

**Figure 5.**
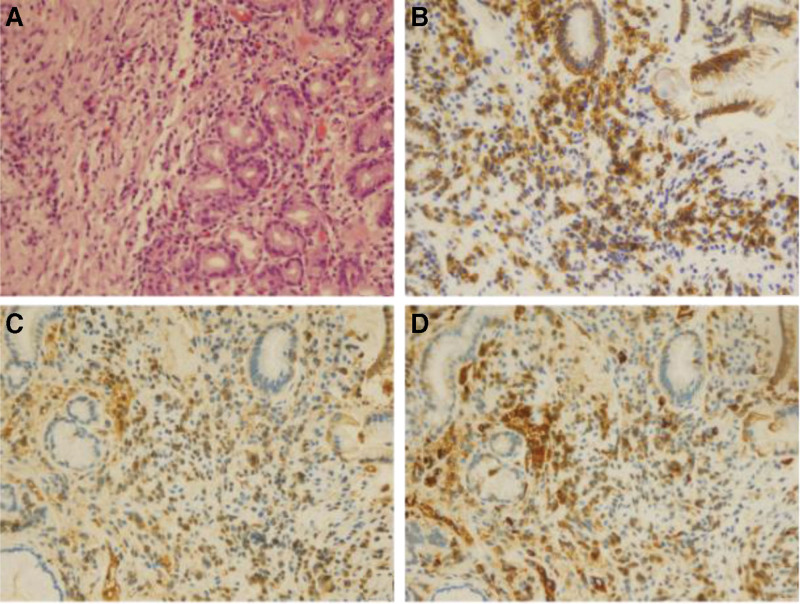
Pathological findings in the gastric cardia. (A) HE staining (×400): massive infiltration of plasma cells, eosinophils, and lymphocytes in the lamina propria of the mucosa. (B) CD138 staining (×400): number of CD138+ plasma cells > 50/HPF. (C) Immunoglobulin G (IgG) staining (×400). (D) IgG4 staining (×400): plasma cell IgG4(+)/IgG(+) cell ratio > 40%. HE = hematoxylin and eosin, HPF = high power field, IgG = immunoglobulin G .

**Figure 6.**
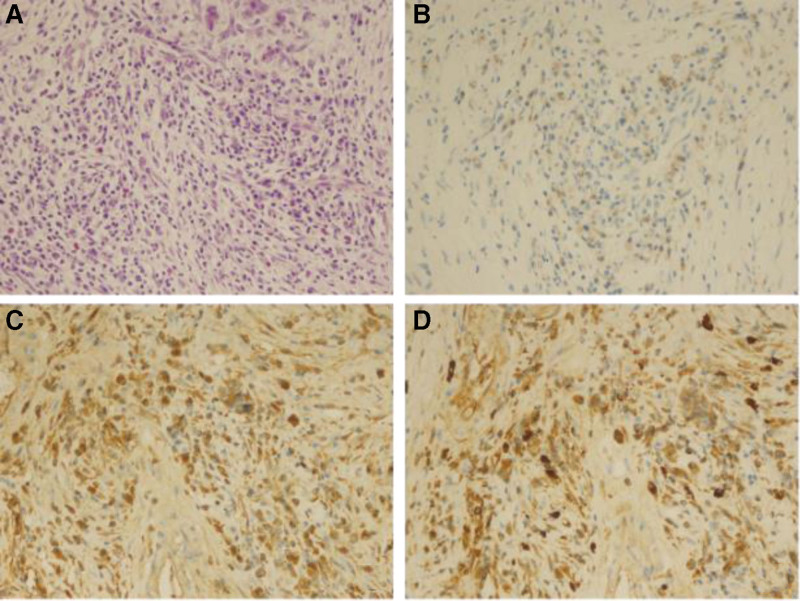
Pathologic findings in the pancreas. (A) HE staining (×400): plasma cells, eosinophils, and lymphocyte infiltration. (B) CD138 staining (×400): CD138+ plasma cells. (C) Immunoglobulin G (IgG) staining (×400). (D) IgG4 staining (×400): IgG4+ plasma cells > 50/HPF, plasma cell IgG4(+)/IgG(+) cell ratio > 40%. HE = hematoxylin and eosin, HPF = high power field, IgG = immunoglobulin G .

**Figure 7.**
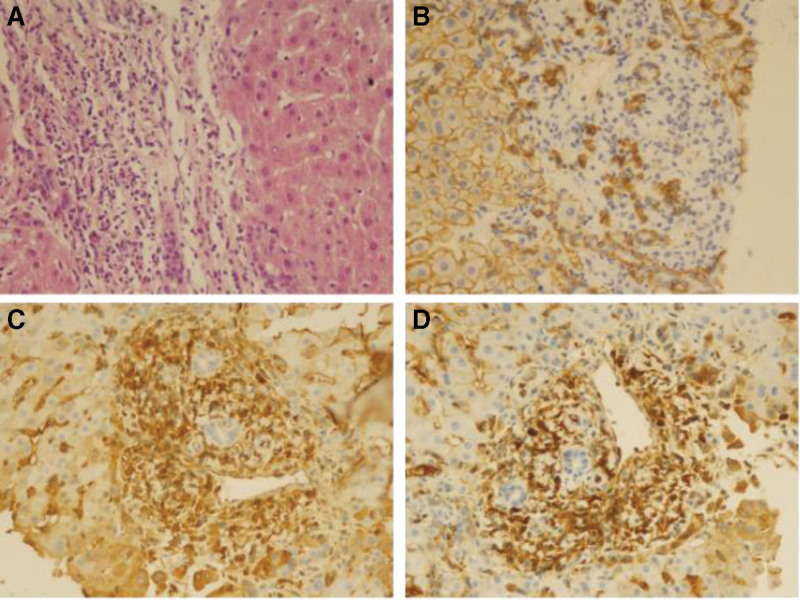
Pathological findings in the liver. (A) HE staining (×400): infiltration of plasma cells, eosinophils, and lymphocytes is visible in the portal tract of liver tissue, with sclerosis around the small bile ducts, and mild interface hepatitis. (B) CD138 staining (×400): the number of CD138+ plasma cells is approximately 50/HPF. (C) Immunoglobulin G (IgG) staining (×400). (D) IgG4 staining (×400): plasma cell IgG4(+)/IgG(+) cell ratio > 40%. HE = hematoxylin and eosin, HPF = high power field.

First physical examination findings revealed the patient’s body temperature was recorded at 36°C, heart rate was 76 beats/min, and blood pressure was 146/81 mm Hg. Physical examination revealed jaundice of the skin and the sclera. The abdomen was flat, with no tenderness, rebound tenderness, or guarding. Shifting dullness was absent, and bowel sounds were normoactive. Following admission, the relevant laboratory tests and examinations were performed. Liver function test results were as follows: ALT 647.0 U/L, AST 384.0 U/L, γ-GT 1455.6 U/L, ALP 742.0 U/L, TBil 66.8 μmol/L, DBil 46.6 μmol/L, and indirect bilirubin (IBil) 20.2 μmol/L (Fig. 2). The serum amylase and lipase levels were 238.0 IU/L and 145.2 U/L, respectively. Multiple tumor markers were assessed, with ferritin and CA 19-9 levels of 644.55 ng/mL and 51.67 U/mL, respectively. To clarify the cause of abnormal liver function, we further refined the following laboratory examinations. Protein electrophoresis revealed a γ-globulin level of 31.32%. The autoimmune liver disease panel was positive for anti-RO52 antibodies, anti-mitochondrial antibodies at a titer of 1:320, and antinuclear antibodies at a titer of 1:320; however, anti-mitochondrial antibody type M2 was negative. Viral hepatitis-related indicators and T-SPOT-TB test results were negative, thereby excluding these etiologies as contributors to liver damage. The patient developed worsening jaundice, pruritus, pale stools, and dark urine. Repeated liver function test results were as follows: ALT 558.0 U/L, AST 467.0 U/L, γ-GT 1346.0 U/L, ALP 1087.0 U/L, TBil 206.4 μmol/L, DBil 162.7 μmol/L, and IBil 43.7 μmol/L (Fig. 2). The tests revealed further deterioration compared with previous results. Contrast-enhanced abdominal computed tomography (CT) findings revealed dilatation of the intrahepatic bile ducts, stenosis of the intrapancreatic segment of the common bile duct, a cyst in the right lobe of the liver, cholecystitis, and right renal atrophy. The patient experienced abdominal pain. Gastroscopy and pathological biopsy were performed to identify the cause of the abdominal pain. Gastroscopy revealed inflammatory changes in the cardia. Gastric fundus elevation, gastric antrum elevation, external gallbladder pressure, and chronic nonatrophic gastritis (Fig. 4). Based on the patient’s history and the above findings, the probability of IgG4-RD was assessed to be high, and gastric, liver, and pancreatic puncture biopsies were performed for histopathological examination. Pathological findings suggested chronic inflammation (acute phase) of the gastric fundic mucosa, characterized by erosions and a massive infiltration of plasma cells, eosinophils, and lymphocytes into the lamina propria of the cardiac mucosa. The number of CD 138+ plasma cells was >50/high power field (HPF) and the plasma cell IgG4(+)/IgG(+) cell ratio was >40% (Fig. 5). The probability of IgG4-RD was determined based on the immunohistochemical findings and clinical presentation. Pathological findings from the pancreatic puncture suggested chronic sclerosing pancreatitis, considering the following parameters: IgG4-association; CD138+ plasma cells, CD38+ plasma cells +, IgG4+ plasma cells > 50/HPF, and plasma cell IgG4(+)/IgG(+) cell ratio > 40% (Fig. 6). The probability of IgG4-RD was determined based on immunohistochemical findings and clinical presentation. Pathological findings of the liver puncture revealed lymphoid, plasma cell, and eosinophilic infiltration in the portal tract of the liver tissue, sclerosis around the small bile ducts, and mild interface hepatitis, consistent with IgG4 cholangitis. The number of CD138+ plasma cells was approximately 50/HPF, and the plasma cell IgG4(+)/IgG(+) cell ratio was >40% (Fig. 7). The probability of IgG4-RD was considered based on the immunohistochemical findings and clinical observation.

Based on the clinical, laboratory, and pathological findings, the patient was diagnosed with IgG4-RD, including IgG4-related pancreatitis (autoimmune pancreatitis), immunoglobulin G4-related sclerosing cholangitis (IgG4-SC), immunoglobulin G4-related autoimmune hepatitis (IgG4-AIH), and immunoglobulin G4-related gastrointestinal disease (IgG4-GID).

Therapy was initiated on March 8, 2022, with oral prednisone acetate (30 mg once daily) in conjunction with acid suppression and calcium supplementation. Following glucocorticoid administration, there was a marked improvement in the skin and sclera jaundice. During follow-up on March 11, 2022, liver function test results were as follows: ALT 281.4 U/L, AST 80.3 U/L, γ-GT 709.8 U/L, ALP 703.0 U/L, TBil 84.4 μmol/L, DBil 67.6 μmol/L, and IBil 416.8 μmol/L; IgG4:17.2 g/L; CA 19-9:32.8 U/mL (Fig. 2). Resolved jaundice, improved liver function tests, reduced IgG4 levels, and the efficacy of glucocorticoid administration further confirmed the diagnosis of IgG4-RD. The patient underwent intermittent outpatient follow-up at our hospital between April 2022 and April 2024. ALT, AST, γ-GT, ALP, TBil, DBil, IBil, serum lipase, and erythrocyte sedimentation rate levels remained within normal limits. Serum amylase levels were maintained between 131 and 203 IU/L, and IgG4 levels were maintained between 5.17 and 8.37 g/L, indicating a significant improvement compared to previous results (Fig. 2). Abdominal MRI performed on April 1, 2022, revealed a cyst in the right lobe of the liver and concurrent stenosis of the intrapancreatic segment of the common bile duct, with improvement noted, as well as right renal atrophy. Follow-up abdominal MRI performed on March 10, 2023, and April 13, 2024, showed no significant changes compared to the findings on April 1, 2022 (Fig. 1). Gastroscopy performed on March 15, 2023, revealed chronic atrophic gastritis (C-2), carditis, disappearance of the gastric antrum, and fundus elevation (Fig. 4). Following glucocorticoid administration, the IgG4-related inflammatory changes in the cardia improved. The significantly enlarged gallbladder decreased in size owing to an improvement in the stenosis of the pancreatic segment of the common bile duct, and the bulge at the gastric antrum caused by external compression of the gallbladder was resolved. The resolution of the fundic elevation was considered to be IgG4-related, and the pathological findings suggested that the inflammatory changes might have been due to the failure to obtain relevant lesions during biopsy. The patient is currently undergoing continuous follow-ups.

## 3. Discussion

IgG4-RD is a chronic fibroinflammatory autoimmune disorder affecting multiple organs. The disease is frequently misdiagnosed or overlooked in clinical practice, potentially due to insufficient awareness among clinicians and the absence of reliable biomarkers. Therefore, clinicians must understand the diagnostic approach to IgG4-RD. Initially, a detailed medical history should be obtained, with a focus on the presence of multi-organ involvement. Subsequently, serum IgG4 testing should be performed, along with exclusionary tests such as antinuclear antibody assays. Further investigations, including CT or MRI, should be tailored to the affected organs for pancreatic or biliary involvement. Finally, a tissue biopsy should be performed to rule out malignancy and confirm the characteristic histopathological features. The presence of IgG4-positive cells alone is insufficient for diagnosing IgG4-RD, and the diagnosis is typically established based on histopathological findings. The comprehensive diagnostic criteria for IgG4-RD can be divided into 3 categories: diffuse or localized swelling or masses in single or multiple organs; serum IgG4 concentrations > 135 mg/dL, and histopathological findings include marked lymphocytic and plasmacytic infiltration and fibrosis, as well as infiltration of IgG4-positive plasma cells in a ratio of IgG4-positive plasma cells/IgG-positive plasma cells > 40% and >10 IgG4-positive plasma cells/HPF.^[5]^ Patients who fulfilled all 3 criteria received a definitive diagnosis, those who fulfilled the first and third criteria received a probable diagnosis, and those who fulfilled the first and second criteria received a probable diagnosis of IgG4-RD.^[5]^ In this case, the patient’s abdominal CT revealed stenosis of the intrapancreatic segment of the common bile duct as well as pancreatic enlargement. Serum IgG4 level was elevated at 21.5 g/L. Pathological findings in the liver, pancreas, and stomach revealed IgG4-positive plasma cell infiltration. Pathological findings of the liver revealed interface hepatitis and sclerosis around the small bile ducts. Given that all 3 diagnostic criteria were met, the patient was definitively diagnosed with IgG4-RD.

IgG4-RD is a systemic condition, and when it affects the pancreas, it manifests as type 1 autoimmune pancreatitis (AIP). Most patients with AIP present with obstructive jaundice as the initial symptom, whereas 40% experience nonspecific upper abdominal pain or discomfort. The patient in our case report presented with nonspecific upper abdominal pain. Based on the abdominal CT findings, elevated serum IgG4 levels, pancreatic biopsy results, and a positive response to glucocorticoid administration, a diagnosis of AIP was established. AIP frequently coexists with IgG4-SC, IgG4-AIH, and other IgG4-RD.^[8]^ IgG4-SC shares clinical features with AIP, including obstructive jaundice, mild upper abdominal discomfort, and abnormal aminotransferase and immunological marker levels; CT findings often reveal pancreatic enlargement. IgG4-AIH was defined as the concurrent presence of the serological, histopathological, and clinical features of both IgG4-RD and AIH. Given its low prevalence, definitive diagnostic criteria for IgG4-AIH remain undefined. However, the diagnostic criteria proposed by Umemura et al^[9]^ are frequently employed, and are as follows: a serum IgG4 level of ≥135 mg/dL; IgG4-positive lymphoplasmacytic cell infiltration of ≥10/HPF upon liver biopsy; and a definitive diagnosis of AIH according to the international acute illness in hepatitis group (IAIHG) score. The patient in our case report exhibited the following characteristics: a serum IgG4 level of 21.5 g/L; significant infiltration of IgG4-positive plasma cells in the liver tissue, and typical AIH diagnosis meeting the diagnostic criteria (simplified IAIHG score of 8) aligned with the diagnostic criteria proposed by Umemura et al. However, whether IgG4-AIH should be considered a subtype of classic AIH or the hepatic involvement of systemic IgG4-RD remains under investigation. Gastrointestinal involvement is rare; therefore, diagnostic criteria remain incompletely defined. Notohara et al^[10]^ defined cases with IgG4-positive cells > 50/HPF and an IgG4-positive/IgG-positive cell ratio > 40% observed in the 3 foci with the most abundant IgG4-positive cells as “possible IgG4-GID.” “Possible IgG4-GID,” further characterized by storiform fibrosis, obliterative phlebitis, and/or perineural inflammation, was defined as “highly suggestive of IgG4-GID.” Based on the current pathological biopsy results of the cardia, this case could only be diagnosed as “possible IgG4-GID.” IgG4-RD has rarely been described in the urinary tract, primarily affecting the kidney and bladder, whereas ureteral cases are extremely rare, often termed “inflammatory pseudotumor” or “idiopathic segmental ureteritis.”^[11,12]^ Marando et al^[12]^ proposed the term “ureteral IgG4-RD” for ureteral lesions exhibiting morphological features similar to those of IgG4-RD in other sites. Preoperative diagnosis of ureteral IgG4-RD is challenging from a clinical and therapeutic perspective, with radiological findings often mimicking those of urothelial carcinoma, leading to misdiagnosis. In this case report, the patient was diagnosed with ureteral inflammatory stricture in 2021. Due to the absence of pathological biopsy and serological IgG4 testing at that time, a definitive diagnosis of ureteral IgG4-RD could not be established. However, placement of a ureteral stent resulted in resolution of the stenosis, with the disappearance of hydroureteronephrosis and the hydroureter (Fig. 3), potentially implicating IgG4-RD involvement of the ureter.

A meta-analysis revealed an elevated overall cancer risk in IgG4-RD patients compared to the general population, with a standardized incidence rate of 2.57. In particular, the risks of pancreatic cancer and lymphoma were significantly higher, with standardized incidence rates of 4.07 and 69.17, respectively.^[13]^ IgG4-RD treatment options include systemic corticosteroids, immunosuppressive drugs, biologics, and surgical removal of affected tissues. Despite these contraindications, corticosteroids remain the first-line treatment for inducing remission.^[6,7]^ Consensus guidelines, both domestic and international, recommend moderate-dose glucocorticoids (0.6–0.8 mg/kg) as initial induction therapy, with treatment response assessment after approximately 1 month.^[4,14,15]^ A dose reduction of 5 mg every 1 to 2 weeks is recommended, with a total treatment duration of 3 to 6 months. In cases where IgG4-RD involves gastric lesions, misdiagnosis and surgical resection are potential outcomes. In our patient, the diagnosis of IgG4-RD was established based on elevated serum IgG4 levels and histopathological findings. The therapeutic effects of the glucocorticoid treatment were significant.

## 4. Conclusions

In summary, we presented a rare case of IgG4-RD involving the pancreas, bile ducts, liver, stomach, and ureter. Clinicians must recognize the diverse manifestations of IgG4-RD, which can affect multiple organs and present various clinical symptoms, potentially leading to missed or incorrect diagnoses. Timely and accurate diagnosis is crucial for appropriate treatment and for avoiding unnecessary surgical interventions. This case report highlights that IgG4-RD should be included in the differential diagnosis of gastric masses and inflammation. A definitive diagnosis can be achieved through biopsy for histopathological evaluation, along with assessment of serum IgG4 levels to guide appropriate therapeutic interventions. Further case reports are needed to elucidate the pathogenesis, clinical characteristics, and criteria for discontinuation of glucocorticoid administration in patients with IgG4-RD.

## Acknowledgments

The authors extend their sincere gratitude to the Departments of Pathology and Radiology at the Affiliated Hospital of Inner Mongolia Medical University for their support in providing the case materials. We also acknowledge the assistance of the clinicians in the collection of data. Special thanks are extended to the patient and their family for their consent and cooperation in the preparation of this case report.

## Author contributions

**Conceptualization:** Jing Qiao, Yinglong Huang.

**Data curation:** Xiaoxia Xu, Ze Wang, Ping Chen, Fene Hao, Yinglong Huang.

**Funding acquisition:** Yinglong Huang.

**Investigation:** Jing Qiao, Xiaoxia Xu, Ze Wang, Fene Hao, Yinglong Huang.

**Methodology:** Jing Qiao, Yinglong Huang.

**Project administration:** Ping Chen, Yinglong Huang.

**Resources:** Xiaoxia Xu, Ping Chen, Yinglong Huang.

**Supervision:** Yinglong Huang.

**Writing – original draft:** Jing Qiao.

**Writing – review & editing:** Yinglong Huang.
